# Supplementary material for: Role and mechanism of NCAPD3 in promoting malignant behaviors in gastric cancer
Source: Front Pharmacol. 2024 Apr 22;15:1341039. doi: 10.3389/fphar.2024.1341039 (PMC11070777; doi:10.3389/fphar.2024.1341039)
Supplement: Supplementary file 11 [file DataSheet2.ZIP › GSEA/Transcipt factor/my_analysis.Gsea.1599461814734/ZNF711_TARGET_GENES.html]

Details for gene set ZNF711\_TARGET\_GENES[GSEA]

|  || Dataset | filtered\_dataset.sample\_info.cls#WT\_versus\_NCAPD3\_MUT |
| Phenotype | sample\_info.cls#WT\_versus\_NCAPD3\_MUT |
| Upregulated in class | WT |
| GeneSet | ZNF711\_TARGET\_GENES |
| Enrichment Score (ES) | 0.24247487 |
| Normalized Enrichment Score (NES) | 1.8542415 |
| Nominal p-value | 0.0028653296 |
| FDR q-value | 0.28627676 |
| FWER p-Value | 0.945 |
Table: GSEA Results Summary

  

Fig 1: Enrichment plot: ZNF711\_TARGET\_GENES      
 Profile of the Running ES Score & Positions of GeneSet Members on the Rank Ordered List

  

| SYMBOL | TITLE | RANK IN GENE LIST | RANK METRIC SCORE | RUNNING ES | CORE ENRICHMENT || 1 | 51111 | KMT5B | 2 | 1.217 | 0.0140 | Yes |
| 2 | 7763 | ZFAND5 | 26 | 1.006 | 0.0087 | Yes |
| 3 | 222236 | NAPEPLD | 36 | 0.977 | 0.0141 | Yes |
| 4 | 9759 | HDAC4 | 46 | 0.925 | 0.0188 | Yes |
| 5 | 388536 | ZNF790 | 52 | 0.913 | 0.0265 | Yes |
| 6 | 80746 | TSEN2 | 53 | 0.909 | 0.0382 | Yes |
| 7 | 7743 | ZNF189 | 74 | 0.851 | 0.0332 | Yes |
| 8 | 2926 | GRSF1 | 75 | 0.849 | 0.0441 | Yes |
| 9 | 92140 | MTDH | 93 | 0.801 | 0.0409 | Yes |
| 10 | 51540 | SCLY | 95 | 0.800 | 0.0504 | Yes |
| 11 | 55006 | TRMT61B | 101 | 0.782 | 0.0564 | Yes |
| 12 | 50861 | STMN3 | 126 | 0.777 | 0.0474 | Yes |
| 13 | 55687 | TRMU | 128 | 0.774 | 0.0565 | Yes |
| 14 | 55781 | RIOK2 | 135 | 0.763 | 0.0615 | Yes |
| 15 | 55900 | ZNF302 | 145 | 0.740 | 0.0639 | Yes |
| 16 | 51174 | TUBD1 | 153 | 0.730 | 0.0677 | Yes |
| 17 | 7733 | ZNF180 | 154 | 0.728 | 0.0770 | Yes |
| 18 | 2186 | BPTF | 159 | 0.717 | 0.0831 | Yes |
| 19 | 54971 | BANP | 172 | 0.707 | 0.0826 | Yes |
| 20 | 23450 | SF3B3 | 178 | 0.697 | 0.0876 | Yes |
| 21 | 23023 | TMCC1 | 181 | 0.695 | 0.0949 | Yes |
| 22 | 55132 | LARP1B | 189 | 0.686 | 0.0982 | Yes |
| 23 | 51018 | RRP15 | 192 | 0.685 | 0.1054 | Yes |
| 24 | 3308 | HSPA4 | 194 | 0.685 | 0.1133 | Yes |
| 25 | 84190 | METTL25 | 195 | 0.684 | 0.1221 | Yes |
| 26 | 4836 | NMT1 | 208 | 0.669 | 0.1212 | Yes |
| 27 | 57521 | RPTOR | 239 | 0.645 | 0.1057 | Yes |
| 28 | 5701 | PSMC2 | 244 | 0.636 | 0.1106 | Yes |
| 29 | 10944 | C11orf58 | 272 | 0.617 | 0.0971 | Yes |
| 30 | 23036 | ZNF292 | 288 | 0.602 | 0.0930 | Yes |
| 31 | 25972 | UNC50 | 297 | 0.593 | 0.0942 | Yes |
| 32 | 2241 | FER | 304 | 0.588 | 0.0970 | Yes |
| 33 | 147929 | ZNF565 | 311 | 0.587 | 0.0998 | Yes |
| 34 | 23211 | ZC3H4 | 314 | 0.586 | 0.1057 | Yes |
| 35 | 84946 | LTV1 | 319 | 0.583 | 0.1100 | Yes |
| 36 | 84135 | UTP15 | 330 | 0.576 | 0.1095 | Yes |
| 37 | 94107 | TMEM203 | 333 | 0.574 | 0.1152 | Yes |
| 38 | 51491 | NOP16 | 334 | 0.573 | 0.1226 | Yes |
| 39 | 92070 | CTBP1-AS2 | 350 | 0.567 | 0.1180 | Yes |
| 40 | 55869 | HDAC8 | 352 | 0.566 | 0.1244 | Yes |
| 41 | 168374 | ZNF92 | 357 | 0.562 | 0.1285 | Yes |
| 42 | 10818 | FRS2 | 363 | 0.558 | 0.1317 | Yes |
| 43 | 57109 | REXO4 | 370 | 0.552 | 0.1340 | Yes |
| 44 | 115509 | ZNF689 | 374 | 0.551 | 0.1387 | Yes |
| 45 | 9786 | KIAA0586 | 379 | 0.547 | 0.1425 | Yes |
| 46 | 10618 | TGOLN2 | 383 | 0.547 | 0.1472 | Yes |
| 47 | 5930 | RBBP6 | 390 | 0.543 | 0.1494 | Yes |
| 48 | 23567 | ZNF346 | 393 | 0.541 | 0.1547 | Yes |
| 49 | 10426 | TUBGCP3 | 402 | 0.535 | 0.1552 | Yes |
| 50 | 83932 | SPRTN | 404 | 0.535 | 0.1613 | Yes |
| 51 | 11019 | LIAS | 409 | 0.533 | 0.1649 | Yes |
| 52 | 56894 | AGPAT3 | 414 | 0.527 | 0.1685 | Yes |
| 53 | 84274 | COQ5 | 419 | 0.523 | 0.1720 | Yes |
| 54 | 57727 | NCOA5 | 425 | 0.517 | 0.1747 | Yes |
| 55 | 5001 | ORC5 | 450 | 0.506 | 0.1622 | Yes |
| 56 | 8607 | RUVBL1 | 451 | 0.506 | 0.1686 | Yes |
| 57 | 23383 | MAU2 | 459 | 0.500 | 0.1695 | Yes |
| 58 | 9690 | UBE3C | 470 | 0.493 | 0.1679 | Yes |
| 59 | 84282 | RNF135 | 473 | 0.489 | 0.1726 | Yes |
| 60 | 80095 | ZNF606 | 479 | 0.487 | 0.1749 | Yes |
| 61 | 4191 | MDH2 | 494 | 0.476 | 0.1699 | Yes |
| 62 | 29101 | SSU72 | 496 | 0.475 | 0.1752 | Yes |
| 63 | 58500 | ZNF250 | 499 | 0.474 | 0.1797 | Yes |
| 64 | 6742 | SSBP1 | 501 | 0.472 | 0.1849 | Yes |
| 65 | 11060 | WWP2 | 503 | 0.471 | 0.1902 | Yes |
| 66 | 1660 | DHX9 | 510 | 0.469 | 0.1914 | Yes |
| 67 | 130502 | TTC32 | 517 | 0.465 | 0.1926 | Yes |
| 68 | 3607 | FOXK2 | 522 | 0.461 | 0.1954 | Yes |
| 69 | 64324 | NSD1 | 534 | 0.455 | 0.1925 | Yes |
| 70 | 3836 | KPNA1 | 548 | 0.448 | 0.1879 | Yes |
| 71 | 1819 | DRG2 | 550 | 0.447 | 0.1929 | Yes |
| 72 | 78988 | MRPL57 | 557 | 0.441 | 0.1938 | Yes |
| 73 | 79707 | NOL9 | 560 | 0.441 | 0.1978 | Yes |
| 74 | 6632 | SNRPD1 | 563 | 0.440 | 0.2019 | Yes |
| 75 | 84335 | AKT1S1 | 572 | 0.437 | 0.2012 | Yes |
| 76 | 9747 | TCAF1 | 575 | 0.436 | 0.2052 | Yes |
| 77 | 93134 | ZNF561 | 590 | 0.431 | 0.1996 | Yes |
| 78 | 4548 | MTR | 592 | 0.428 | 0.2043 | Yes |
| 79 | 55181 | SMG8 | 602 | 0.422 | 0.2026 | Yes |
| 80 | 80824 | DUSP16 | 610 | 0.418 | 0.2024 | Yes |
| 81 | 8021 | NUP214 | 611 | 0.417 | 0.2077 | Yes |
| 82 | 7290 | HIRA | 612 | 0.417 | 0.2131 | Yes |
| 83 | 55308 | DDX19A | 615 | 0.416 | 0.2168 | Yes |
| 84 | 5147 | PDE6D | 624 | 0.410 | 0.2157 | Yes |
| 85 | 9852 | EPM2AIP1 | 634 | 0.406 | 0.2138 | Yes |
| 86 | 26065 | LSM14A | 636 | 0.405 | 0.2182 | Yes |
| 87 | 79807 | GSTCD | 644 | 0.402 | 0.2178 | Yes |
| 88 | 22929 | SEPHS1 | 645 | 0.401 | 0.2229 | Yes |
| 89 | 56252 | YLPM1 | 665 | 0.394 | 0.2129 | Yes |
| 90 | 79832 | QSER1 | 675 | 0.389 | 0.2108 | Yes |
| 91 | 9453 | GGPS1 | 677 | 0.389 | 0.2150 | Yes |
| 92 | 5134 | PDCD2 | 678 | 0.388 | 0.2200 | Yes |
| 93 | 149076 | ZNF362 | 680 | 0.388 | 0.2241 | Yes |
| 94 | 55750 | AGK | 697 | 0.380 | 0.2163 | Yes |
| 95 | 84159 | ARID5B | 699 | 0.379 | 0.2204 | Yes |
| 96 | 155435 | RBM33 | 706 | 0.373 | 0.2204 | Yes |
| 97 | 55958 | KLHL9 | 710 | 0.370 | 0.2228 | Yes |
| 98 | 6651 | SON | 716 | 0.365 | 0.2235 | Yes |
| 99 | 23350 | U2SURP | 719 | 0.364 | 0.2266 | Yes |
| 100 | 6182 | MRPL12 | 729 | 0.359 | 0.2241 | Yes |
| 101 | 134430 | WDR36 | 733 | 0.359 | 0.2263 | Yes |
| 102 | 26054 | SENP6 | 739 | 0.356 | 0.2269 | Yes |
| 103 | 51000 | SLC35B3 | 740 | 0.355 | 0.2314 | Yes |
| 104 | 6605 | SMARCE1 | 749 | 0.349 | 0.2296 | Yes |
| 105 | 440275 | EIF2AK4 | 753 | 0.347 | 0.2316 | Yes |
| 106 | 6241 | RRM2 | 755 | 0.346 | 0.2353 | Yes |
| 107 | 90120 | C9orf69 | 758 | 0.343 | 0.2381 | Yes |
| 108 | 27300 | ZNF544 | 759 | 0.343 | 0.2425 | Yes |
| 109 | 9238 | TBRG4 | 786 | 0.330 | 0.2261 | No |
| 110 | 79590 | MRPL24 | 800 | 0.320 | 0.2199 | No |
| 111 | 5901 | RAN | 810 | 0.313 | 0.2168 | No |
| 112 | 51575 | ESF1 | 812 | 0.309 | 0.2199 | No |
| 113 | 8904 | CPNE1 | 814 | 0.308 | 0.2231 | No |
| 114 | 26608 | TBL2 | 821 | 0.304 | 0.2222 | No |
| 115 | 26121 | PRPF31 | 825 | 0.301 | 0.2237 | No |
| 116 | 3146 | HMGB1 | 831 | 0.295 | 0.2235 | No |
| 117 | 2224 | FDPS | 841 | 0.273 | 0.2199 | No |
| 118 | 23435 | TARDBP | 842 | 0.272 | 0.2234 | No |
| 119 | 4691 | NCL | 843 | 0.270 | 0.2268 | No |
| 120 | 3312 | HSPA8 | 848 | 0.244 | 0.2268 | No |
| 121 | 5723 | PSPH | 859 | -0.267 | 0.2223 | No |
| 122 | 50640 | PNPLA8 | 881 | -0.296 | 0.2095 | No |
| 123 | 27230 | SERP1 | 892 | -0.306 | 0.2054 | No |
| 124 | 27131 | SNX5 | 907 | -0.333 | 0.1986 | No |
| 125 | 3716 | JAK1 | 939 | -0.363 | 0.1787 | No |
| 126 | 8881 | CDC16 | 963 | -0.383 | 0.1654 | No |
| 127 | 55186 | SLC25A36 | 969 | -0.387 | 0.1664 | No |
| 128 | 10793 | ZNF273 | 980 | -0.394 | 0.1635 | No |
| 129 | 115825 | WDFY2 | 994 | -0.400 | 0.1583 | No |
| 130 | 51646 | YPEL5 | 1014 | -0.413 | 0.1485 | No |
| 131 | 11138 | TBC1D8 | 1026 | -0.424 | 0.1452 | No |
| 132 | 9926 | LPGAT1 | 1036 | -0.434 | 0.1437 | No |
| 133 | 5889 | RAD51C | 1059 | -0.451 | 0.1320 | No |
| 134 | 100534599 | ISY1-RAB43 | 1082 | -0.462 | 0.1205 | No |
| 135 | 317649 | EIF4E3 | 1102 | -0.479 | 0.1116 | No |
| 136 | 4194 | MDM4 | 1117 | -0.492 | 0.1068 | No |
| 137 | 55370 | PPP4R1L | 1124 | -0.497 | 0.1084 | No |
| 138 | 84918 | LRP11 | 1129 | -0.501 | 0.1117 | No |
| 139 | 7444 | VRK2 | 1145 | -0.509 | 0.1063 | No |
| 140 | 146057 | TTBK2 | 1148 | -0.513 | 0.1113 | No |
| 141 | 92014 | SLC25A51 | 1155 | -0.519 | 0.1132 | No |
| 142 | 1983 | EIF5 | 1219 | -0.595 | 0.0708 | No |
| 143 | 4189 | DNAJB9 | 1223 | -0.598 | 0.0761 | No |
| 144 | 22931 | RAB18 | 1231 | -0.608 | 0.0784 | No |
| 145 | 1788 | DNMT3A | 1253 | -0.641 | 0.0699 | No |
| 146 | 8412 | BCAR3 | 1261 | -0.648 | 0.0727 | No |
| 147 | 8916 | HERC3 | 1269 | -0.660 | 0.0756 | No |
| 148 | 54464 | XRN1 | 1301 | -0.703 | 0.0600 | No |
| 149 | 23365 | ARHGEF12 | 1329 | -0.751 | 0.0483 | No |
| 150 | 3329 | HSPD1 | 1402 | -1.183 | 0.0063 | No |
Table: GSEA details [plain text format]

  

Fig 2: ZNF711\_TARGET\_GENES      
 Blue-Pink O' Gram in the Space of the Analyzed GeneSet

  

Fig 3: ZNF711\_TARGET\_GENES: Random ES distribution      
 Gene set null distribution of ES for **ZNF711\_TARGET\_GENES**
